# Comprehensive Analysis of Receptor Status, Histopathological Classifications (B1–B5), and Cumulative Histological Dimensions in Breast Cancer: Predictors of Malignancy and Diagnostic Implications

**DOI:** 10.3390/cancers16203471

**Published:** 2024-10-14

**Authors:** Oana Maria Burciu, Ioan Sas, Adrian-Grigore Merce, Simona Cerbu, Aurica Elisabeta Moatar, Anca-Elena Eftenoiu, Ionut Marcel Cobec

**Affiliations:** 1 Doctoral School, Faculty of Medicine, “Victor Babes” University of Medicine and Pharmacy Timisoara, 300041 Timisoara, Romania; 2 Department of Functional Sciences, Medical Informatics and Biostatistics Discipline, “Victor Babes” University of Medicine and Pharmacy, 300041 Timisoara, Romania; 3 Department of Obstetrics and Gynecology, “Victor Babes” University of Medicine and Pharmacy, 300041 Timisoara, Romania; 4 Department of Cardiology, Institute of Cardiovascular Diseases, 300310 Timisoara, Romania; 5 Discipline of Radiology and Medical Imaging, “Victor Babes” University of Medicine and Pharmacy Timisoara, 300041 Timisoara, Romania; 6 ANAPATMOL Research Center, Faculty of Medicine, “Victor Babes” University of Medicine and Pharmacy Timisoara, 300041 Timisoara, Romania; 7 Clinic of Internal Medicine-Cardiology, Klinikum Freudenstadt, 72250 Freudenstadt, Germany; 8 Department of Medical Genetics, “Carol Davila” University of Medicine and Pharmacy, 050474 Bucharest, Romania; 9 Clinic of Obstetrics and Gynecology, Klinikum Freudenstadt, 72250 Freudenstadt, Germany

**Keywords:** breast cancer, histopathological classification, estrogen receptor, progesterone receptor, androgen receptor

## Abstract

**Simple Summary:**

The aim of this study was to assess the impact of various factors—such as age, age at menarche, age at first childbirth, number of births, body mass index (BMI), living environment (rural vs. urban), and other factors later discussed—on the likelihood of developing breast cancer. This article’s original research was based on data gathered from 687 breast biopsies, which were analyzed through comparative analysis and regression analysis to identify possible predictors for malignancy and tumor size. The comparative analysis and regression models in this study provide deeper insights into the roles of various variables, particularly positive hormonal receptor status, in malignancy.

**Abstract:**

Introduction: Breast cancer has become one of the most serious and widespread public health concerns globally, affecting an increasing number of women—and, in rare cases, men—across the world. It is the most common cancer among women across all countries. In this study, we aimed to evaluate the influence of demographic factors, medical and reproductive history, diagnostic techniques, and hormone receptor status on the development and progression of breast cancer. Materials and Methods: A total of 687 female patients from Romania underwent standard breast examination techniques, including clinical breast examination, mammography, ultrasonography, and, ultimately, breast biopsy. Statistical analysis was performed using the R programming language and RStudio software. The study included a comparative analysis and a prediction analysis for malignancy and tumor size (cumulative histological dimension) through logistic and linear regression models. Results: The comparative analysis identified several variables associated with malignancy: older age (*p* < 0.001), non-vulnerability (*p* = 0.04), no daily physical activity (*p* = 0.002), no re-biopsy (*p* < 0.001), immunohistochemistry use (*p* < 0.001), use of larger gauge needles (*p* < 0.001), ultrasound-guided biopsy (*p* < 0.001), and vacuum biopsy (*p* < 0.001). The hormone receptor statuses—estrogen receptor (ER), progesterone receptor (PR), and androgen receptor (AR)—showed statistically significant differences in distribution across breast cancer B classifications. Logistic regression analysis identified ER, PR, and age as significant predictors of malignancy. Linear regression analysis revealed histopathological results, living environment, geographical region, vulnerability, prior breast examination, and the number of histological fragments as significant predictors of cumulative histological dimension. Conclusions: Our predictive models demonstrate the impact of demographic factors, medical history, diagnostic techniques, and hormone receptor status on breast cancer development and progression, accounting for a significant portion of the variance in malignancy and cumulative histological dimension.

## 1. Introduction

Breast cancer is responsible for about 30% of the cancers affecting women, and it accounts for 11.6% of all new cases of cancer for both men and women. Male breast cancer cases are rare, representing less than 1%, and are treated approximately in the same manner with some clinical differences [1,2,3].

The number of women diagnosed each year is in constant growth, with recent studies reporting increases in incidence rates of approximately 1.44% per year since 1990 and others reflecting an increase of about 0.5% annually [4,5]. Part of this growth can be attributed to advancements in screening programs, improved technology, and, to some extent, more awareness of the online environment [6]. Years of scientific research have uncovered intriguing correlations between several modifiable and non-modifiable factors and their role in developing breast cancer.

Female sex is perhaps the first risk factor taken into consideration due to enhanced estrogen and progesterone levels in women (vs. men), which have been linked to a higher probability of developing breast and ovarian malignancies in various studies [7].

Age is a highly significant risk factor, and possibly the most important one, as numerous studies from around the world have demonstrated that older age is associated with a higher chance of developing a malignant breast lesion. The median age of developing breast cancer has been stated to be around 60–61 years old [8,9,10].

Various modifiable and non-modifiable factors that may influence the development of malignant breast lesions—such as genetic mutations, hormone replacement therapy, a sedentary lifestyle, high body mass index (BMI), moderate-to-high alcohol intake, and others—have been extensively studied and discussed in numerous research articles [11,12].

Approximately 5–10% of all breast cancers are caused by genetic mutations, most commonly in the BRCA1 and BRCA2 genes, which can also cause ovarian and pancreatic cancers. Similar to most breast tumors, ovarian cancers are typically estrogen-dependent, relying on estrogen for their development and subsequent progression [13,14].

From a histological perspective, malignant breast lesions are broadly classified into two main categories, namely in situ carcinomas and invasive carcinomas. In situ, carcinoma is confined to the breast ducts or lobules, with abnormal cells proliferating within these structures without invading surrounding breast tissue. In contrast, invasive carcinoma extends beyond the ducts and lobules, infiltrating the surrounding connective tissue. The most common subtypes are ductal carcinoma in situ (DCIS), lobular carcinoma in situ (LCIS), and their invasive counterparts—ductal invasive carcinoma and lobular invasive carcinoma. Additionally, several other histological subtypes exist, including in situ variants such as comedo, cribriform, and papillary carcinoma, and invasive variants such as mixed invasive ductal and lobular carcinoma, tubular carcinoma, and others. The two most common types of invasive carcinoma are invasive ductal carcinoma (IDC), which stands for approximately 80% of all breast cancers, followed by invasive lobular carcinoma (ILC), accounting for approximately 10–15% of cases [15,16].

Compared to IDC, ILC is typically characterized by positive hormone receptor status—estrogen receptor (ER) and progesterone receptor (PR), but negative human epidermal growth factor (HER2) expression, which generally leads to a favorable response to endocrine therapy. Additionally, ILC is more likely to exhibit multifocality and multicentricity, and its detection by mammography can be more challenging, often resulting in diagnosis at a later stage. ILC has also been associated with a higher recurrence rate compared to IDC after 5–10 years, with unusual metastatic sites [17].

In order to evaluate a biopsy fragment from a histopathological point of view, specialists use the B classification, which ranges from B1 to B5b. B1 represents normal breast tissue with no evidence of malignancy, B2 indicates benign lesions (such as fibrocystic changes or fibroadenomas), B3 is assigned to lesions of uncertain malignant potential, and B4 corresponds to suspicious lesions that require further diagnostic investigation. The B5 category is further divided into B5a, which indicates non-invasive carcinoma in situ, and B5b, representing invasive carcinoma, the most aggressive and malignant form. When a lesion is classified as B3, the typical clinical management involves re-biopsy, either through vacuum-assisted or surgical methods, rather than observation, due to the uncertainty associated with B3 lesions. A B4 lesion necessitates further histopathological and immunohistochemical staining to establish a definitive diagnosis. B5b lesions, characterized by more aggressive histological features, generally require a combination of chemotherapy, surgery, and radiotherapy, aligned with accurate TNM staging (tumor size, lymph node involvement, and metastasis). In contrast, B5a lesions, which have a more limited spread, can often be treated with surgery ensuring clear margins, followed by radiotherapy. Therefore, determining whether a tumor is invasive is crucial for guiding appropriate therapeutic management [18].

To assess if breast cancer is hormone-dependent or receptor-negative, a thorough analysis of tissue samples using immunohistochemistry is essential. This allows the breast pathologist to accurately evaluate the hormone receptor status of the tumor. It is estimated that 70–80% of breast cancers are hormone-dependent, characterized by the overexpression of ER, PR, or HER2, which promote tumor growth. In clinical terms, receptor positivity in breast tumors is generally associated with a more favorable prognosis, as these tumors can be treated with adjuvant hormonal therapy, which is effective in reducing tumor size and limiting its capacity to spread. In addition to these receptors, the androgen receptor (AR) plays a complex role in breast cancer pathophysiology, acting as both a promoter and an inhibitor of tumor cell growth under certain conditions of estrogen stimulation. ARs are expressed in 60–90% of breast cancers, usually in ER-positive tumors [19,20,21,22].

An ER-positive cancer cell can receive growth signals from estrogen, whereas a PR-positive cancer cell responds to signals from progesterone. However, it might seem like a disadvantage; hormone-dependent or hormone receptor-positive breast tumors generally have a better prognosis and higher 5-year overall survival rates compared to receptor-negative tumors, largely due to the effectiveness of adjuvant hormone therapy, as previously mentioned. Conversely, breast cancers that lack ER, PR, and Her2 receptors are classified as triple-negative. These tumors are unresponsive to endocrine therapies and are often linked to poorer overall survival, as they tend to follow a more aggressive course, leading to earlier recurrences and higher rates of metastasis [23,24].

By targeting the estrogen signaling pathway, endocrine therapy has become a powerful aid to surgical treatment for breast cancer or can act as a standalone treatment. Recent research has demonstrated that in the metastatic stage of hormone receptor-positive, HER2-negative breast cancer, combined treatment with CDK4/6 inhibitors and other agents can lead to extended progression-free survival [25,26,27,28].

Breast cancer is a significant health concern for women worldwide, and while every aspect previously discussed is crucial, it is essential to emphasize that actively promoting breast cancer screening should be a primary focus. Relevant studies affirmed that early breast cancer detection through screening programs can lead to an average 23% mortality risk reduction among women in high-income countries [29,30].

The best and most accessible and cost-efficient methods to screen for breast cancer are imagistic methods, such as mammography, ultrasound, and breast MRI, and their derivates. Clinical breast examination can represent an option if the previously mentioned imagistic methods are not available but are considered less reliable due to a degree of subjectivity [31].

The objective of this study was to evaluate the influence of demographic factors, medical and reproductive history, diagnostic techniques, and hormone receptor status on the development and progression of breast cancer and identify key predictors of malignancy and tumor size (cumulative histological dimension) in breast lesions, with a particular focus on understanding their diagnostic implications.

## 2. Materials and Methods

### 2.1. Study Population

The study population comprised women aged 50 to 69 years, with no personal history of breast cancer, residing in urban and rural areas of the West (W), North-East (NE), North-West (NW), and South-East (SE) regions of Romania. Between 2020 and 2023, participants first consulted a breast specialist and underwent mammography. If a suspicious lesion was detected, a follow-up breast ultrasound was performed. If the lesion remained suspicious on ultrasound, a biopsy was scheduled. To ensure accuracy, all imaging procedures were reviewed by two experienced breast imagers. In total, 687 biopsies were collected.

To evaluate the biopsied fragments from a histopathological perspective, the B classification system was used (B indicating biopsy). This classification ranges from B1 to B5b and is distinct from the BI-RADS (Breast Imaging Reporting and Data System) score, which ranges from 0 to 5 and is employed by radiologists to describe lesions identified on mammograms and assess the need for breast biopsy. It is important to note that the histological fragments included in our study originated from tumors classified as BI-RADS 4 or 5.

In this study conducted on 687 female patients who underwent biopsies for breast tumors, we performed a comprehensive analysis of various factors associated with breast cancer, focusing on receptor statuses, histopathological classifications (B1–B5), and cumulative histological dimensions. Several numerical and categorical variables were analyzed to compare the characteristics of participants with malignant and benign conditions. The numerical variables included age, BMI, age at menarche, age at first birth, and the number of births. Age was recorded in years to capture the range of ages at diagnosis. BMI was calculated based on participants’ height and weight to assess body composition. The age at menarche, which marks the onset of menstruation, and the age at first birth were both recorded, as reproductive history plays a significant role in breast cancer risk. Additionally, the number of live births was documented as an important reproductive factor.

The categorical variables covered a broad range of demographic, lifestyle, medical, and diagnostic factors. Participants were classified by their living environment, distinguishing between urban and rural settings, which may reflect differences in lifestyle and healthcare access. Region was another key variable, with participants categorized into four geographic areas: North-East (NE), North-West (NW), South-East (SE), and West (W), to explore regional variations in breast cancer risk.

A key variable was vulnerability status, which classified participants as either vulnerable or non-vulnerable. Vulnerability was defined using a composite index that considered women who are self-employed in agriculture, those currently or previously in foster care, and women who have exited the child protection system. Additionally, homeless women, women of Roma ethnicity, and women from single-parent families were also considered vulnerable. Other factors contributing to vulnerability included addiction to alcohol, drugs, or other toxic substances, as well as being victims of domestic violence or human trafficking. This operational definition ensured consistency in how the vulnerability was assessed. Participants’ breastfeeding status was also documented, noting whether they had breastfed and, if so, for how long, with durations categorized into less than 1 month, 1–6 months, 6–12 months, or more than 12 months.

Daily physical activity was another important lifestyle variable. It was defined based on recommendations from health organizations, with participants classified as engaging in physical activity if they reported at least 30 min of moderate exercise per day. Alcohol consumption, red meat consumption, and smoking status were also recorded as lifestyle factors, with participants categorized based on whether they engaged in these behaviors.

In terms of medical and reproductive history, several variables were analyzed. Hormonal treatment during menopause was documented to assess whether participants had used hormone therapies, such as estrogen-only or combined estrogen-progesterone treatments. The study also recorded whether participants had a family history of cancer, focusing on breast and ovarian cancers, which are known to be linked to an increased risk of breast cancer. Menopause status was categorized as no menopause, early, normal, or late menopause, reflecting the role of hormonal changes in breast cancer risk. Additionally, participants’ history of prior breast examinations and breast interventions was included, providing insight into their previous medical care.

Finally, the study documented various diagnostic and biopsy techniques. A re-biopsy was noted for participants who required a second biopsy following the initial procedure. The use of immunohistochemistry was critical for determining hormone receptor status, including ER, PR, and AR, as these markers guide treatment decisions. The type of biopsy pistol used (single-use automatic, reusable automatic, or semi-automatic) and the needle thickness (gauge sizes ranging from 9 to 16) were also recorded, depending on the clinical requirements for tissue sampling. The system type used during the biopsy, whether ultrasound- or mammography-guided, was documented, along with whether participants underwent vacuum-assisted biopsies or surgical biopsies.

### 2.2. Statistical Analysis

In this study, the statistical analysis was conducted to evaluate both continuous and categorical variables. Continuous variables, which did not follow a normal distribution as assessed by the Shapiro-Wilk test (*p* < 0.05 indicating non-Gaussian distribution), were presented as medians and interquartile ranges (IQRs). Categorical variables were summarized as frequencies and proportions. Due to the non-normal distribution of the continuous variables, non-parametric tests were employed throughout the analysis.

The Mann–Whitney U test was applied to compare the differences between the two groups for continuous variables. For categorical variables, Pearson’s Chi-Square test was used to compare proportions. The level of association between categorical variables was assessed using Cramer’s V, where effect sizes were classified as follows: <0.05 = tiny, 0.05 to 0.1 = very small, 0.1 to 0.2 = small, 0.2 to 0.3 = medium, 0.3 to 0.4 = large, and ≥0.4 = very large.

To identify potential risk factors for malignancy, a logistic regression analysis was conducted. The selection of variables was performed using the backward elimination method, and the best model was chosen based on the Akaike Information Criterion (AIC). The performance of the model was evaluated using Receiver Operating Characteristic (ROC) parameters and Nagelkerke’s R^2^. The significance of risk factors was assessed using Wald’s test. A multiple linear regression analysis was employed to identify independent predictors of cumulative histological dimension. Again, the backward elimination method and AIC were used to select the best model, while model performance was assessed using the adjusted R^2^. The *p*-values for predictors were derived from the Student’s *t*-test.

The results were presented in both graphical and tabular forms. All data processing and statistical analysis were conducted using the R programming language (R Core Team, 2023) and the latest version of RStudio software (Posit, 2023). A *p*-value < 0.05 was considered statistically significant, with 95% confidence intervals (CI) reported where appropriate.

## 3. Results

### 3.1. Comparative Analysis of Demographic, Lifestyle, and Clinical Factors between Malignant and Benign Breast Lesions

**Figure 1:**
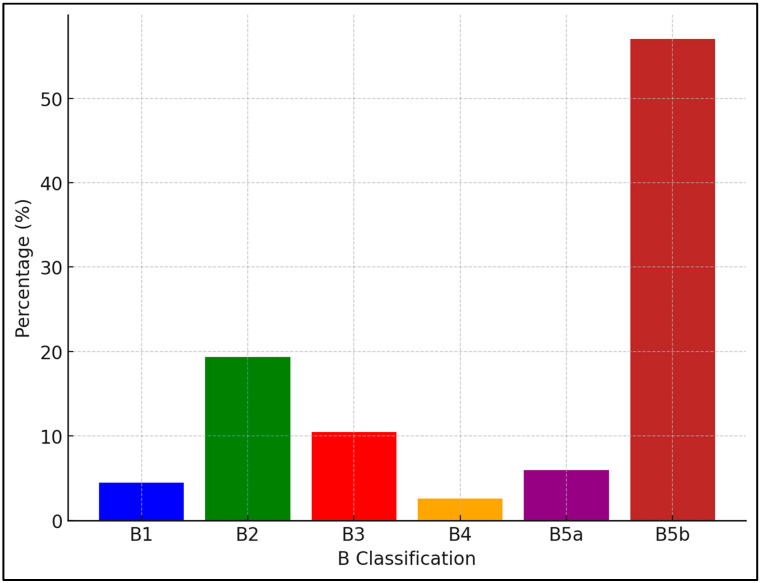
Distribution of Cases Across B Classification. X-axis—B Classification, Y-axis—the percentage of cases within each group.

Figure 1 illustrates the distribution of cases across the B classification (B1, B2, B3, B4, B5a, and B5b). Each bar depicts the proportion of total cases for each respective classification. In terms of distribution, B5b was the most dominant category, with over 50% of the cases. Following B5b, B2 had the second-largest proportion, contributing to around 20% of the cases, while B3 accounted for about 10% of the cases. B1, B4, and B5a categories represented smaller segments of the data, with B1 accounting for approximately 5%, B4 contributing the least at around 2–3%, and B5a slightly exceeding B4.

**Figure 2:**
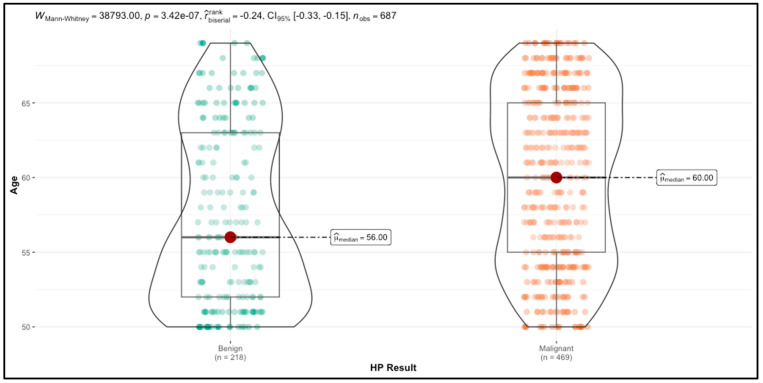
Comparison of Age Distribution Between Benign and Malignant Breast Lesions.

Table 1 presents the comparative analysis results of numerical variables between malignant and benign breast lesions, while Figure 2 illustrates the age differences between the two groups. Women diagnosed with breast cancer were older (median age 60 years) compared to those with benign conditions (median age 56 years), and this difference was statistically significant (*p* < 0.001). However, other variables, including BMI, age at menarche, age at first birth, and the number of births, did not exhibit statistically significant differences between the two groups. The lack of significance for these variables suggests that, in this sample, reproductive and lifestyle factors such as BMI, menarche age, first birth age, and number of births may not strongly differentiate between malignant and benign breast cancer cases. Therefore, age appears to be the most prominent demographic factor associated with malignancy in this dataset, while other factors do not show a clear association.

**Figure 3:**
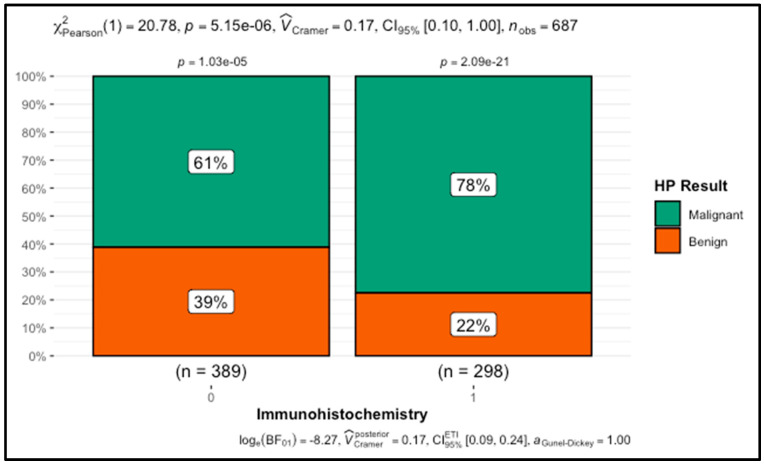
Comparison Between Immunohistochemistry Use and Histopathological Outcome (Malignant vs. Benign). The y-axis represents the proportions of cases. Green bars indicate malignant cases, and orange bars indicate benign cases. The groups are classified as 1 for the immunohistochemistry group and 0 for the non-immunohistochemistry group.

Table 2 and Figure 3 present a comparison of demographics, lifestyle, medical history, and diagnostic techniques between malignant and benign breast lesion cases. Regarding living environment, a slightly higher percentage of malignant cases were observed in urban areas (71%) compared to rural areas (64%), although this difference was not statistically significant (*p* = 0.07). Similarly, regional distribution did not show significant differences in malignancy rates, with patients from the NE, NW, SE, and W regions showing comparable proportions of malignant and benign cases (*p* = 0.23).

Vulnerability status, however, was significantly associated with malignancy, with a higher proportion of malignancy in non-vulnerable patients (71%) compared to vulnerable patients (64%) (*p* = 0.04). No significant associations were found between breastfeeding status or breastfeeding duration and malignancy. Daily physical activity was significantly associated with malignancy, with less active patients (72%) being more likely to have malignant cases compared to those who reported regular activity (60%) (*p* = 0.002). Alcohol consumption, red meat consumption, and smoking did not show statistically significant associations with malignancy.

In terms of medical and reproductive history, neither hormonal treatment during menopause, family medical history of cancer, menopause status, prior breast examination, nor prior breast intervention were significantly associated with malignancy.

On the diagnostic side, several variables were strongly associated with malignancy. Patients who did not undergo a re-biopsy were more likely to have malignant results (72% malignant vs. 28% benign) compared to the re-biopsy group (6% malignant vs. 94% benign) (*p* < 0.001). Similarly, cases where immunohistochemistry was performed had a significantly higher likelihood of malignancy (78%) compared to those where it was not (61%) (*p* < 0.001). Additionally, patients biopsied with larger gauge needles (14-gauge or larger) had a significantly higher rate of malignancy (*p* < 0.001), and ultrasound-guided biopsy was also strongly associated with malignant outcomes (72% vs. 28% benign), while mammography-guided biopsy showed a reverse trend, with more benign cases (*p* < 0.001). Similarly, vacuum biopsy was significantly associated with a higher proportion of benign cases, while non-vacuum biopsy methods showed a greater proportion of malignancies (*p* < 0.001). Finally, surgical biopsy showed no significant difference in the malignancy rates compared to non-surgical biopsies (*p* = 0.15).

Table 3 describes the distribution of ER, PR, and AR statuses across the B classification. For ER status, it was observed that most ER-positive cases are concentrated in the higher B categories, particularly B5b, where 89% of the cases are ER-positive. Lower categories (B1 to B4) showed significantly lower percentages of ER positivity, with only 1–5% of cases being ER-positive in these categories. In contrast, ER-negative cases were more prevalent in the lower categories, with 43% of B2 cases and 10% of B1 cases being ER-negative, and this decreased significantly in B5b, where only 15% of cases were ER-negative. Cases in higher B categories were more likely to be ER-positive, whereas lower categories had a greater proportion of ER-negative cases (*p* < 0.001).

Similarly, for PR status, the distribution was skewed toward higher B categories for PR-positive cases, with 91% of B5b cases being PR-positive. Lower B categories (B1 to B4) had considerably fewer PR-positive cases, ranging from 0 to 4%. Conversely, PR-negative cases were more common in the lower B categories, with 39% of B2 cases and 9% of B1 cases being PR-negative. The proportion of PR-negative cases was notably lower in B5b (21%). This comparative analysis showed that PR positivity was predominantly seen in higher categories (especially B5b), while PR negativity was more common in lower B categories (*p* < 0.001).

For AR status, the comparison revealed a different pattern. While AR-positive cases were predominantly concentrated in the B5b category, with 74% of B5b cases being AR-positive, AR positivity was notably absent in B4 and B5a classifications, and only a small proportion of B2 and B3 cases were AR-positive (9% and 17%, respectively). In contrast, AR-negative cases were more evenly distributed across the B classification but still showed higher percentages in the lower categories (B1, B2, and B3). The AR-negative cases were also present in B5b, though to a lesser extent (56%). The comparative analysis showed that while AR positivity is highly concentrated in B5b, its presence across the other classifications was more variable compared to ER and PR (*p* < 0.001).

In summary, the comparative analysis revealed that ER and PR positivity were significantly more prevalent in the higher-risk B5b category, starkly contrasting with the lower B categories, where ER and PR-negative cases predominated. In the case of AR status, the pattern was less consistent, though AR positivity was primarily seen in the B5b category.

### 3.2. Logistic Regression Analysis and Performance Evaluation of Predictors for Malignancy in Breast Lesions

The logistic regression analysis presented in Table 4 identified key predictors of malignancy in breast lesions. ER positivity was the strongest predictor, with an odds ratio (OR) of 15.79 and a 95% CI of 6.09–50.71, indicating that ER-positive patients were over 15 times more likely to have malignant breast cancer compared to ER-negative patients (*p* < 0.001). Similarly, PR positivity was also a significant predictor, with an OR of 4.83 (CI: 1.38–15.23), showing that PR-positive patients were almost five times more likely to have malignancy (*p* = 0.009). Age was another significant factor, with an OR of 1.06 (CI: 1.02–1.11), meaning that the likelihood of malignancy increases by 6% for each additional year of age (*p* = 0.005). The overall model’s explanatory power, as indicated by Nagelkerke’s R^2^, was 0.533, suggesting that the model explains approximately 53% of the variance in malignancy.

### 3.3. Linear Regression Analysis of Predictors for Cumulative Histological Dimension in Breast Lesions

In the absence of a direct measure of tumor size, the cumulative histological dimension was used as an indirect yet reliable proxy for estimating tumor dimensions in this study. The cumulative histological dimension represents the total size of the tissue fragments obtained during biopsy, which correlates with the tumor’s physical extent. Given that larger tumors typically require more extensive sampling, this measure serves as a practical alternative for estimating tumor size when exact measurements are unavailable. The use of cumulative histological dimensions allows for meaningful analysis of tumor characteristics and their relationship with various clinical and demographic factors, making it a suitable surrogate for tumor size in the context of breast cancer research.

The linear regression analysis presented in Table 5 identified several predictors for the cumulative histological dimension in breast lesions. The histopathological result showed a positive and significant association, with an estimate of 3.82 (CI: 1.20–6.43), indicating that a positive histopathological result is associated with an increase in cumulative histological dimension (*p* = 0.004).

Interestingly, living in an urban environment was associated with a significant decrease in cumulative histological dimension, with an estimate of −12.18 (CI: −20.76–3.60, *p* = 0.005). Similarly, the regions NW, SE, and W were all associated with substantial negative estimates, indicating smaller cumulative histological dimensions for patients in these regions. Region W had the largest reduction, with an estimate of −34.76 (CI: −38.49–31.02, *p* < 0.001).

On the other hand, non-vulnerable patients showed a significant increase in cumulative histological dimension, with an estimate of 12.16 (CI: 3.65–20.66, *p* = 0.005), indicating that vulnerability status may play a role in the size of the histological dimension. Having had a prior breast examination was associated with a small but significant decrease in cumulative histological dimension (estimate = −2.77, CI: −5.20–0.34, *p* = 0.025).

Finally, the number of histological fragments was a strong positive predictor, with each additional fragment being associated with a 2.99 increase in cumulative histological dimension (CI: 2.66–3.31, *p* < 0.001), making it one of the most significant predictors in the model. The adjusted R^2^ value of 0.485 indicates that the model explains approximately 48.5% of the variance in the cumulative histological dimension, reflecting a moderately strong model.

## 4. Discussion

### 4.1. Demographic Factors and Patient History

In the current study, older age was associated with malignancy (median age 60 for breast cancer vs. median age 56 benign lesions) and represented a predictor of malignancy, consistent with findings from other research [32]. In other words, the likelihood of developing breast cancer increases by 6% for each additional year of age, as indicated by our regression analysis. An interesting and comparable study from the University of Oxford stated each 5-year acceleration in biological age corresponded with a 15% increase in breast cancer risk in women [33].

Lack of daily physical activity was significantly associated with malignancy in our patient cohort. This observation has also been highlighted in other studies concerning breast cancer in postmenopausal women [34]. Similarly, other works pointed out that little or no physical activity may lead to an alteration in sex and metabolic hormones, leading to increased oxidative stress and inflammation in the body, facilitating the onset of breast cancer [35].

### 4.2. Diagnostic Tools, Hormone Receptor Status, and B Classification

US-guided biopsies and larger gauge needles yielded more malignant results, while mammography-guided and vacuum-assisted biopsies yielded more benign results. These associations were statistically significant. Additionally, we observed a higher occurrence of malignant lesions in patients who did not undergo a re-biopsy. In the literature, various biopsy techniques have been compared in terms of accuracy, and one study reported no statistically significant difference regarding US-guided biopsy with 14, 16, or 18-gauge needles [36]. Survival for different biopsy methods has also been investigated in other studies showing higher mortality risk associated with core needle biopsy (CNB) compared to fine needle aspiration (FNA) in women who did not undergo radiotherapy, probably due to a higher risk of tumor seeding [37].

When analyzing hormone receptor status distribution across the previously discussed B classification, we found that both PR and ER positivity were predominantly observed in higher categories (particularly B5b), while PR negativity was more common in lower categories. A similar trend was seen with AR-positive cases, whereas AR-negative cases were more evenly distributed. The distribution of ER, PR, and AR across the B classification emphasizes the distinct hormone receptor profiles associated with different B categories in breast cancer, with all findings showing strong statistical significance. Logistic regression analysis identified ER positivity as the strongest predictor of malignancy, increasing the risk of developing cancerous breast disease by 15 times. PR positivity also represented a significant predictor, increasing the risk by approximately 5 times. The carcinogenic role of estrogen has been previously studied and discussed; one study emphasized the importance of diet and exercise in lowering endogenous estrogen levels to reduce the risk of breast cancer [7,38].

### 4.3. Tumor Size and Extent

In the absence of the exact tumor size, for our research, we used cumulative histological dimension, representing the total size of tissues collected during biopsy, in order to approximate the dimensions of the tumor. As anticipated, the cumulative histological dimension correlated with a positive (cancerous) histopathological result. However, a significant and unexpected finding revealed that an urban living environment was associated with a decrease in cumulative histological dimensions, suggesting that patients from urban areas have smaller cumulative histological dimensions compared to rural patients. This trend was also observed in patients from the NW, SE, and W regions, all of which were negative predictors of cumulative histological dimension. Other studies have also investigated tumor size differences between women from rural and urban areas, reaching similar conclusions [39,40,41]. Additional predictors of cumulative histological dimension identified in this study were prior clinical breast examination, which served as a negative predictor, and the number of histological fragments, as expected, a positive predictor.

A study showed a clear correlation between lymph node involvement and tumor size. Furthermore, lymph node involvement was more prevalent in patients with tumors larger than 5 cm and individuals with a positive histological diagnosis of invasive ductal carcinoma [42].

### 4.4. Limitations

The significance of BRCA mutations has been well established as a major genetic risk factor for developing breast cancer. However, due to data limitations in our cohort, BRCA status was not available for analysis in this study but will be a key focus for our future research. Further investigation into epigenetic factors and their implication in breast cancer risk should be pursued as part of a more personalized approach to cancer prevention and treatment. Additionally, the potential role of oral contraceptives in breast cancer development will be a key focus of future research.

## 5. Conclusions

Our study has revealed, through statistical analysis, intriguing associations between various variables and their impact on the risk of developing breast cancer. Some of these findings have been discussed in previous research; however, we believe that our work provides updated insights. The comparative analysis presented in this study shows strong associations between various variables and malignancy. The regression models explain a significant portion of the variance in malignancy and cumulative histological dimension, respectively. The final model for malignancy identified ER status, PR status, and age as significant predictors. In contrast, the model for cumulative histological dimensions highlighted several factors, including histological results, living environment, geographic region, vulnerability, prior breast examination, and number of histological fragments. In conclusion, our analysis highlights the significant roles of demographic factors, medical and reproductive history, diagnostic techniques, and hormone receptor status in the development and progression of breast cancer.

## Figures and Tables

**Table 1:** Comparison of Numerical Variables Between Malignant and Benign Breast Lesions.

Variable	Malignant	Benign	*W*	*p*-Value
Age (years)	60.00 (55.00–65.00)	56.00 (52.00–63.00)	38,793.00	<0.001
BMI (kg/m^2^)	29.27 (25.71–32.87)	28.73 (25.77–32.00)	48,144.50	0.22
Menarche (years)	14.00 (13.00–14.00)	14.00 (13.00–14.75)	53,030.50	0.41
First birth (years)	22.00 (21.00–25.00)	22.00 (20.00–24.00)	47,515.50	0.13
No. births	3.00 (2.00–3.00)	3.00 (2.00–3.00)	50,463.50	0.77

Abbreviations: BMI—Body Mass Index, *W*—Mann–Whitney U statistic, *p*-value—test result for Mann–Whitney U test.

**Table 2:** Comparison of Demographic, Lifestyle, Medical History, and Diagnostic Techniques Between Malignant and Benign Breast Lesions.

	Variable	Category	Malignant	Benign	*p*-Value
Demographic and Lifestyle Factors	Living Environment	Urban	307 (71%)	127 (29%)	0.07
		Rural	162 (64%)	91 (36%)	
	Region	NE	148 (72%)	58 (28%)	0.23
		NW	229 (67%)	112 (33%)	
		SE	18 (55%)	15 (45%)	
		W	74 (69%)	33 (31%)	
	Vulnerability	Yes	170 (64%)	97 (36%)	0.04
		No	299 (71%)	121 (29%)	
	Breastfeeding	Yes	432 (68%)	205 (32%)	0.37
		No	37 (74%)	13 (26%)	
	Breastfeeding period	<1 month	23 (77%)	7 (23%)	0.18
		1–6 months	226 (70%)	99 (30%)	
		6–12 months	126 (63%)	75 (37%)	
		>12 months	94 (72%)	37 (28%)	
	Daily Physical Activity	Yes	123 (60%)	82 (40%)	0.002
		No	346 (72%)	136 (28%)	
	Alcohol Consumption	Yes	35 (73%)	13 (27%)	0.47
		No	434 (68%)	205 (32%)	
	Red Meat Consumption	Yes	358 (67%)	175 (33%)	0.25
		No	111 (72%)	43 (28%)	
	Smoking	Yes	79 (70%)	34 (30%)	0.68
		No	390 (68%)	184 (32%)	
Medical and Reproductive History	Hormonal Treatment during Menopause	Yes	24 (63%)	14 (37%)	0.49
		No	445 (69%)	204 (31%)	
	FMH Cancer	Yes	36 (73%)	13 (27%)	0.42
		No	433 (68%)	205 (32%)	
	Menopause Status	No	44 (56%)	34 (44%)	0.10
		Early	78 (70%)	33 (30%)	
		Normal	332 (70%)	142 (30%)	
		Late	15 (62%)	9 (38%)	
	Prior Breast Examination	Yes	193 (67%)	94 (33%)	0.63
		No	276 (69%)	124 (31%)	
	Prior Breast Intervention	Yes	28 (78%)	8 (22%)	0.21
		No	441 (68%)	210 (32%)	
Diagnostic and Biopsy Techniques	Re-biopsy	Yes	2 (6%)	33 (94%)	<0.001
		No	467 (72%)	184 (28%)	
	Immunohistochemistry	Yes	231 (78%)	67 (22%)	<0.001
		No	238 (61%)	151 (39%)	
	Biopsy Pistol Type	Single-use automatic	68 (64%)	38 (36%)	0.47
		Reusable automatic	388 (69%)	172 (31%)	
		Semi-automatic	13 (62%)	8 (38%)	
	Needle Thickness	9-gauge	11 (29%)	27 (71%)	<0.001
		12-gauge	22 (61%)	14 (39%)	
		14-gauge	435 (71%)	177 (29%)	
		16-gauge	1 (100%)	0 (0%)	
	System Type	Ultrasound-guided	447 (72%)	177 (28%)	<0.001
		Mammography-guided	22 (35%)	41 (65%)	
	Vacuum Biopsy	Yes	19 (33%)	38 (67%)	<0.001
		No	450 (71%)	180 (29%)	
	Surgical Biopsy	Yes	38 (60%)	25 (40%)	0.15
		No	431 (69%)	193 (31%)	

Abbreviations: *p*-value—test result for Pearson’s Chi-Square test, FMH—family medical history, NE—North-East, NW—North-West, SE—South-East, W—West.

**Table 3:** Distribution of Estrogen, Progesterone, and Androgen Receptor Status Across the B Classification in Breast Cancer.

Receptor	Type	B1	B2	B3	B4	B5a	B5b	*p*-Value
ER	+	0 (0%)	4 (1%)	14 (4%)	3 (1%)	21 (5%)	348 (89%)	<0.001
	−	31 (10%)	129 (43%)	58 (20%)	15 (5%)	20 (7%)	44 (15%)	
PR	+	0 (0%)	4 (1%)	12 (3%)	2 (1%)	15 (4%)	324 (91%)	<0.001
	−	31 (9%)	129 (39%)	60 (18%)	16 (5%)	26 (8%)	68 (21%)	
AR	+	0 (0%)	4 (9%)	8 (17%)	0 (0%)	0 (0%)	34 (74%)	<0.001
	−	31 (5%)	129 (20%)	64 (10%)	18 (3%)	41 (6%)	358 (56%)	

Abbreviations: ER—Estrogen Receptors, PR—Progesterone Receptors, AR—Androgen Receptors, +—Positive, − —Negative, *p*-value—result of Pearson Chi-Square test.

**Table 4:** Logistic Regression Analysis of Predictors for Malignancy in Breast Lesions.

Predictors	OR	CI	*p*-Value
ER [+]	15.79	6.09–50.71	<0.001
PR [+]	4.83	1.38–15.23	0.009
Age	1.06	1.02–1.11	0.005
$R^{2}$ Nagelkerke = 0.533			

Abbreviations: ER—Estrogen Receptors, PR—Progesterone Receptors, +—Positive, OR—Odds Ratio, CI—95% Confidence Interval, *p*-value—Wald’s test results.

**Table 5:** Linear Regression Analysis of Predictors for Cumulative Histological Dimension in Breast Lesions.

Predictors	Estimates	CI	*p*-Value
Histopathological result [+]	3.82	1.20–6.43	0.004
Living Environment [Urban]	−12.18	−20.76–−3.60	0.005
Region [NW]	−12.15	−14.92–−9.38	<0.001
Region [SE]	−10.41	−16.24–−4.57	<0.001
Region [W]	−34.76	−38.49–−31.02	<0.001
Vulnerability [No]	12.16	3.65–20.66	0.005
Breast Examination [Yes]	−2.77	−5.20–−0.34	0.025
No. histological fragments	2.99	2.66–3.31	<0.001
$R^{2}$ adjusted = 0.485			

Abbreviations: +—Positive, CI—Confidence Interval, *p*-value—Student’s t-test result, NW—North-West, SE—South-East, W—West.

## Data Availability

Further information concerning the present article is available from the corresponding author upon reasonable request.
